# Manifold Topological Deep Learning for Biomedical Data

**DOI:** 10.21203/rs.3.rs-6149503/v1

**Published:** 2025-04-07

**Authors:** Xiang Liu, Zhe Su, Yongyi Shi, Yiying Tong, Ge Wang, Guo-Wei Wei

**Affiliations:** 1 Department of Mathematics, Michigan State University, MI, 48824, USA; 2 Biomedical Imaging Center, Rensselaer Polytechnic Institute, NY, 12180, USA; 3 Computer Science and Engineering, Michigan State University, MI 48824, USA; 4 Department of Electrical and Computer Engineering, Michigan State University, MI 48824, USA; 5 Department of Biochemistry and Molecular Biology, Michigan State University, MI 48824, USA

**Keywords:** Biomedical Data Analysis, Differentiable Manifold, Hodge Decomposition, Topological Deep Learning

## Abstract

Recently, topological deep learning (TDL), which integrates algebraic topology with deep neural networks, has achieved tremendous success in processing point-cloud data, emerging as a promising paradigm in data science. However, TDL has not been developed for data on differentiable manifolds, including images, due to the challenges posed by differential topology. We address this challenge by introducing manifold topological deep learning (MTDL) for the first time. To highlight the power of Hodge theory rooted in differential topology, we consider a simple convolutional neural network (CNN) in MTDL. In this novel framework, original images are represented as smooth manifolds with vector fields that are decomposed into three orthogonal components based on Hodge theory. These components are then concatenated to form an input image for the CNN architecture. The performance of MTDL is evaluated using the MedMNIST v2 benchmark database, which comprises 717,287 biomedical images from eleven 2D and six 3D datasets. MTDL significantly outperforms other competing methods, extending TDL to a wide range of data on smooth manifolds.

## 1 Introduction

Topological deep learning (TDL) is an emerging field that integrates topological methods with deep learning techniques to perform learning tasks such as regression, classification, and representation learning [1]. Unlike traditional black-box deep learning models, TDL models offer greater interpretability by leveraging topological features and representations to explicitly capture the underlying geometric and structural properties of data. Since its introduction in 2017 [2], TDL has rapidly evolved, leading to a diverse set of methods and models. Besides its methodological advances, TDL has been successfully applied in various domains, including biology, chemistry, materials science, neuroscience, and social networks [3, 4]. Current TDL models mainly focus on combinatorial data structures, such as point clouds and graphs. Compared to combinatorial data, differentiable manifold data contains richer geometric information and is more suitable for analysis using methods from differential topology, such as differential forms and differential operators. These methods enable the study of continuous, smooth phenomena that cannot be adequately captured through purely combinatorial approaches. Despite this advantage, there are currently no TDL models designed for differentiable manifold data.

Differentiable manifolds, such as curves and surfaces, are ubiquitous in real-world data. For example, DNA chains, object surfaces, and images are all natural examples of differentiable manifolds. Thus, extending TDL to differentiable manifold data is both meaningful and necessary. However, two primary challenges hinder this extension: firstly, although the images are inherently manifold data, it is nontrivial to rigorously model them as differentiable manifolds while preserving essential differentiable and topological properties. Secondly, designing an efficient model that combines the mathematical methods from differential topology with deep learning poses theoretical and computational challenges.

With the advancements in Topological Data Analysis [5, 6], particularly the remarkable successes achieved by persistent homology [7], several studies have employed topological methods for manifold data analysis. For instance, using simplicial complexes or cubical complexes to model images [8] and using curves or knots to represent amino acid chains [9], then computing persistent homology for data analysis. While these methods are powerful in capturing the topological structures of manifold data across various scales, they exhibit limitations in capturing the smooth differentiable information within the manifold data, which can be addressed by incorporating methods from differential topology, such as vector fields, differential forms, and differential operators. Moreover, although differentiable manifolds have been utilized in manifold topological learning [10], such as in modeling protein-ligand complexes [11], these methods have not yet been extended to deep learning architectures.

Recently, a discrete topology-preserving Hodge theory for differentiable manifolds embedded in Cartesian grids has been introduced [12] and successfully applied to single-cell RNA velocity analysis [13]. This theory provides an efficient way for modeling images as differentiable manifolds since images are naturally embedded in Cartesian grids. On the other hand, the MedMNIST v2 dataset offers a standard and reliable benchmark for evaluating model performance in medical image classification. The MedMNIST v2 dataset contains twelve 2D datasets and six 3D datasets, covering major medical data modalities, the data scale ranges from 100 to 100000, and the task type includes binary, multi-class classification, ordinal regression, and multi-label classification, making it highly suitable for assessing the efficiency, robustness, and generalizability of models [14].

Here, we introduce, for the first time, a Manifold Topological Deep Learning (MTDL) Model use the de Rham-Hodge theory, a landmark of the 20th Century’s mathematics. MTDL integrates the discrete Hodge theory from differential topology, the Transformer encoder architecture, and convolutional operations, providing a novel framework for extending TDL to differentiable manifold data. In the MTDL model, the input image is represented as a discrete differentiable manifold and a vector field defined on this manifold. The Hodge Laplacian theory is then employed to decompose the vector field into three orthogonal components: curl-free, divergence-free, and harmonic parts. These components are concatenated to form a new image representation, which is passed to the CNN architecture for the prediction task. We evaluate MTDL on the MedMNIST v2 dataset, including 717,287 images from eleven 2D datasets and six 3D datasets. MTDL significantly outperforms other models, establishing MTDL as an efficient framework for TDL on differentiable manifold data.

## 2 Results

### 2.1 Overview of MTDL

**Figure 1:**
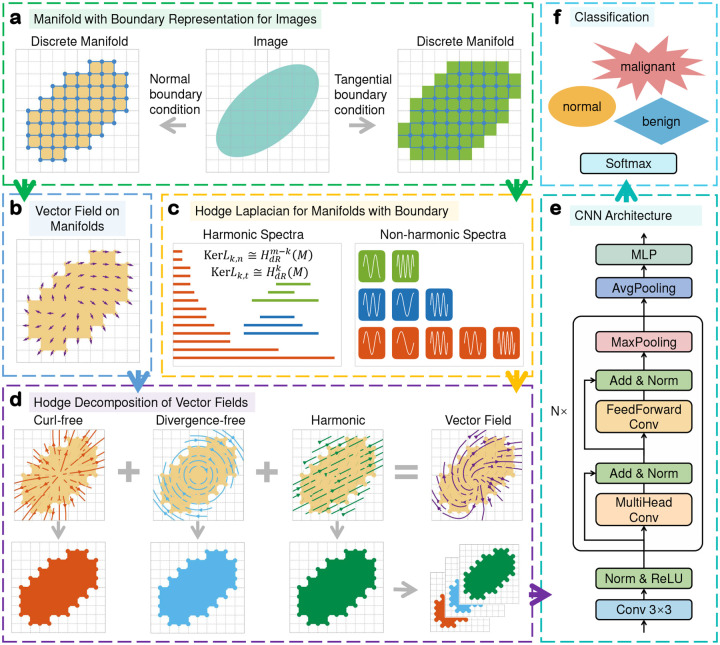
Model architecture of MTDL. The original image is first modeled as a discrete manifold on Cartesian grids under specific boundary conditions (**a**). A vector field is then constructed on the manifold (**b**). Using the discrete Hodge Laplacian for manifolds with boundary (**c**), this vector field is decomposed into three orthogonal components: curl-free, divergence-free, and harmonic parts (**d**). These components are subsequently concatenated to form a multi-channel image, which serves as the input of CNN for the classification task (**e**).

The discrete Hodge theory on Cartesian grids provides an approach for decomposing an image into three distinct components, each capturing different geometric and topological features. To leverage this theory in TDL, we propose a MTDL model that integrates discrete Hodge theory, Transformer encoder architecture, and convolutional operations for image classification. The architecture of MTDL is illustrated in Fig. 1. As shown in the figure, the original image is first represented as a discrete manifold on Cartesian grids under normal or tangential boundary conditions (Fig. 1a). This manifold representation establishes a mathematical formalization of the images, serving as the groundwork for further analysis using Hodge theory, such as using the harmonic spectra of Hodge Laplacian to detect the loop structures of the manifold (Fig. 1c). Subsequently, a vector field that encodes the image information is constructed on the discrete manifolds (Fig. 1b). There are several methods for constructing vector fields from images (Supplementary Information), each method provides a specific perspective on the image’s content and structure. The generated vector field is then decomposed into three orthogonal components through the Hodge decomposition. These components, including curl-free, divergence-free, and harmonic parts, are concatenated to form a multi-channel representation of the decomposed images (Fig. 1d). Finally, the resulting representation is fed into the CNN for image classification (Fig. 1e). This CNN is based on the Transformer encoder architecture by adding a maxpooling operation and replacing the multihead attention and feedforward layers with convolution operations.

### 2.2 Evaluation of MTDL

#### 2.2.1 Dataset

The MedMNIST v2 dataset [14] is an updated version of the original MedMNIST dataset [15]. It is an MNIST-like collection of standardized biomedical images comprising twelve 2D datasets and six 3D datasets that cover primary medical imaging modalities, such as X-ray, Optical Coherence Tomography (OCT), Ultrasound, Computed Tomography (CT), Electron Microscope, and Magnetic Resonance Angiography (MRA). These datasets support a wide range of classification tasks, including binary classification, multi-class classification, ordinal regression, and multi-label classification. The data sizes range from 100 to 100,000 samples. In total, MedMNIST v2 includes 708,069 2D images and 9,998 3D images, with standard train-validation-test splits provided for all datasets.

Among these datasets, BreastMNIST2D is derived from a dataset of 780 breast ultrasound images [16]. The original dataset has been reported to contain certain inconsistencies that could significantly impact model performance [17]. To ensure the validity and reliability of our evaluation, we exclude this dataset and utilize the remaining eleven 2D datasets along with all six 3D datasets for assessing our model’s performance. The image resolutions we used are 224×224 for 2D images and 64×64×64 for 3D images. Further details about the datasets can be found in the Supplementary Information.

#### 2.2.2 Evaluation Protocols

We use the MedMNIST v2 split training and validation sets to train and select hyperparameters and report the results of the test set. Accuracy (ACC) and Area Under the ROC Curve (AUC) are used as evaluation metrics to ensure a fair comparison with benchmark methods reported in the literature [14, 18, 19, 20, 21, 22, 23]. To enhance the reliability of the results, we repeated the process three times with different random seeds and use the average value as the final performance of our model.

#### 2.2.3 Overall Performance

**Figure 2:**
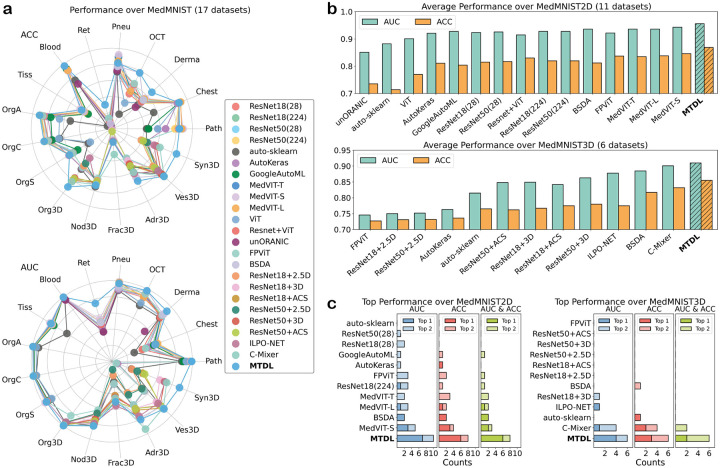
Performance comparison between MTDL model and other models on the MedMNIST v2 dataset. **a**: Comparison of model performance in terms of AUC and ACC across all 17 datasets of MedMNIST v2. The polygon representing the MTDL model covers the largest area, indicating its superior performance compared to the other models. **b**: Average performance of all models over 2D and 3D tasks. MTDL consistently achieves higher AUC and ACC values, outperforming all other models for both types of tasks. **c**: Frequency of top-ranking performance across 2D and 3D tasks. MTDL significantly surpasses all other models, demonstrating its consistent superiority in both 2D and 3D tasks.

Performance comparison of the proposed MTDL model with other state-of-the-art methods on the MedMNIST v2 dataset, in terms of AUC and ACC, is presented in Fig. 2 (detailed values refer to Supplementary). Two radar charts are used to show the performance comparison among different models across all 17 datasets for ACC and AUC respectively. As shown in the figure, the polygon corresponding to MTDL model covers the largest area and is situated at the outermost edge of the region occupied by the polygons of all the models, demonstrating its superior overall performance for medical image classification (Fig. 2a). Notably, MTDL demonstrates significant improvements over the second-best models in specific datasets:

- DermaMNIST: AUC improves from 0.937 to 0.962, and ACC improves from 0.780 to 0.836.
- RetinaMNIST: AUC improves from 0.773 to 0.874, and ACC improves from 0.568 to 0.655.
- OrganMNIST3D: AUC improves from 0.995 to 0.999, and ACC improves from 0.912 to 0.952.
- SynapseMNIST3D: AUC improves from 0.866 to 0.951, and ACC improves from 0.820 to 0.931.

Additionally, we compute the average AUC and ACC separately for 2D and 3D datasets, MTDL consistently outperforms all other models for both 2D and 3D tasks (Fig. 2b). Specifically, for 2D datasets, MTDL achieves an average AUC of 0.956 and an ACC of 0.868, outperforming the second-best model, which achieves an average AUC of 0.943 and an ACC of 0.846. For 3D datasets, MTDL achieves an average AUC of 0.910 and an ACC of 0.855, compared to the second-best model’s average AUC of 0.901 and ACC of 0.832.

Furthermore, we count the frequency of top performance for all models. As shown in the figure, MTDL can outperform all other models in AUC and ACC for both 2D and 3D tasks (Fig. 2c). Specifically, for 2D tasks, MTDL achieves the highest AUC and ACC on six tasks, including RetinaMNIST (1,600 samples), DermaMNIST (10,015 samples), BloodMNIST (17,092 samples), OrganCMNIST (23,660 samples), OrganAMNIST (58,850 samples), and OCTMNIST (109,309 samples). This demonstrates its ability to perform effectively on prediction tasks of varying data scales. When considering the top-2 models, MTDL ranks within the top 2 in a frequency of 10/11 for AUC, 8/11 for ACC, and 8/11 for both AUC and ACC, which is significantly better than the second-best model, which ranks within top 2 in 5/11 for AUC, 4/11 for ACC, and 3/11 for both AUC and ACC. For 3D tasks, MTDL ranks best in both AUC and ACC for 2 tasks while no other model achieves the top rank for both metrics on any dataset. Moreover, MTDL ranks in the top 2 with a frequency of 6/6 for AUC, 6/6 for ACC, and 6/6 for both ACU and ACC, compared with the second-best model’s performance of 4/6 for AUC, 4/6 for ACC, and 2/6 for both AUC and ACC.

These results highlight the overall superiority of MTDL in comparison to other state-of-the-art models, demonstrating its effectiveness in handling both 2D and 3D medical image classification tasks.

#### 2.2.4 Robustness Analysis Across Data Modality, Scale, and Task Type

To assess the robustness and generalizability of model performance, we divide the 17 datasets into groups based on data modality, data scale, and task type (refer to Supplementary Information), and then compare the average performance of all models within each group. To ensure a fair comparison, for each group, MTDL is evaluated only against models that reported results for all datasets in the respective group.

**Figure 3:**
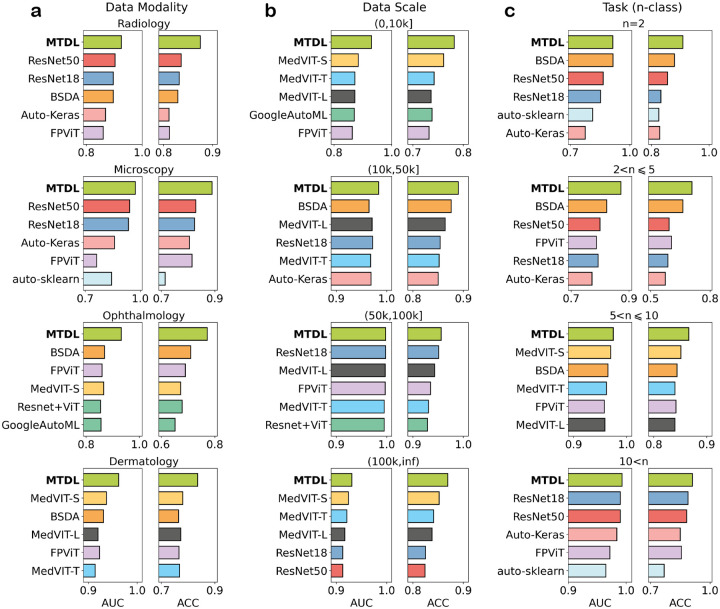
Performance comparison between MTDL and other models on different groups based on data modality, data scale, and task type. Here we only show the best six models for each group. **a**: Comparison on four data modality groups (Radiology, Microscopy, Ophthalmology, Dermatology). **b**: Comparison on four data scale groups (n<10K,10K⩽n<50K,50K⩽n<100K,100K<n) where n is the sample numbers of each dataset. **c**: Comparison on four task type groups (n=2,2<n⩽5,5<n⩽10,10<n) where n is the class number of each dataset.

For data modality, we divide the datasets into four groups: Radiology (X-ray, CT, MRA), Microscopy (Pathology, Electron Microscope), Ophthalmology, and Dermatology. The performance comparison between MTDL and other models is shown in Fig. 3a. It can be seen that MTDL consistently outperforms all other models in both AUC and ACC across all groups. Specifically, MTDL achieves an average AUC (ACC) of 0.926 (0.875) for Radiology, compared to the second-best model’s performance of 0.903 (0.836). For Microscopy, MTDL obtains an average AUC (ACC) of 0.973 (0.890) while the second-best model achieves a score of 0.942 (0.829). For Ophthalmology, MTDL gets an average AUC (ACC) of 0.932 (0.772), significantly surpassing the second-best model’s score of 0.869 (0.710). For Dermatology, MTDL obtains an average AUC (ACC) of 0.962 (0.836), compared to the second-best model’s performance of 0.937 (0.780). Note that MTDL can maintain an AUC above 0.930 for all four groups and an ACC above 0.835 for groups except Ophthalmology, the ACC for Ophthalmology is slightly smaller than other groups. We attribute this to the RetinaMNIST dataset within the Ophthalmology group since this dataset only contains 1600 samples. Despite this, MTDL still significantly outperforms other models for this group.

For data scale, we divide the datasets into four groups based on the sample size n of each dataset: G1 (n⩽10K), G2 (10K<n⩽50K), G3 (50K<n⩽100K), and G4 (100K<n). The performance in terms of AUC and ACC for all models is presented in Fig. 3b. MTDL also ranks best in both metrics for all groups. Specifically, MTDL achieves an average AUC (ACC) of 0.930 (0.782) for G1, compared to the second-best model’s performance of 0.884 (0.761). For G2, MTDL scores 0.984 (0.890) compared to the second-best model’s score of 0.965 (0.876). In G3, both MTDL and the second-best model achieve an average AUC of 0.998, but MTDL has a slightly higher ACC (0.956 vs. 0.951). For G4, MTDL obtains 0.931 (0.869) compared to the second-best model’s 0.924 (0.852). MTDL can get an AUC exceeding 0.930 for all four groups and an ACC exceeding 0.860 for groups except G1, this is reasonable because bigger data usually leads to better performance.

For task type, we divide the datasets into four groups based on the number of classes n for each classification task: G1 (n=2), G2 (2<n⩽5), G3 (5<n⩽10), and G4 (10<n). The performance for all models is shown in Fig. 3c. MTDL again achieves the best overall performance. Specifically, MTDL achieves an average AUC (ACC) of 0.914 (0.909) for G1 compared to the second-best model’s performance of 0.915 (0.880). For G2, MTDL scores 0.872 (0.709), significantly surpassing the second-best model’s score of 0.823 (0.663). For G3, MTDL obtains 0.975 (0.866) compared to the second-best model’s 0.970 (0.851). In G4, MTDL attains an average AUC (ACC) of 0.993 (0.911) compared to the second-best model’s value of 0.990 (0.882). MTDL achieves strong performance for G1, G3, and G4, with AUC exceeding 0.910 and ACC exceeding 0.880. We think the slightly lower performance on G2 is due to the inclusion of the small-sized RetinaMNIST dataset within this group.

These results demonstrate the superiority, robustness, and generalizability of MTDL across various data scales, data modalities, and task types, indicating its great potential for medical image analysis. It is noteworthy that MTDL has only 0.56M parameters for 2D tasks and 0.75M parameters for 3D tasks, which is significantly smaller than models such as ResNet, GoogleNet, Vision Transformer (ViT), and MedViT. Despite its lightweight architecture, MTDL demonstrates exceptional performance.

#### 2.2.5 Evaluation on Clinical Data

In MedMNIST v2, most datasets are derived from clinical sources, that is, human subjects treated in hospitals and medical centers, such as the German National Center for Tumor Diseases [24], Zhongshan Hospital Affiliated to Fudan University [14], and Guangzhou Women and Children’s Medical Center [25], among others. The majority of source datasets are simply processed through center-cropping and resizing to uniform dimensions for inclusion in MedMNIST v2. Consequently, models based on MedMNIST v2 are generally reliable.

To better understand the clinical applicability of our model for medical image analysis, we need to check the effects of image resizing process on model performance. We utilized the HAM10000 dataset, the original clinical dataset for DermaMNIST, and evaluated the performance of MTDL on it. HAM10000 consists of 10015 dermatoscopic images from different populations, including a representative collection of all important diagnostic categories in the realm of pigmented lesions. Over 50% of lesions in it have been confirmed by pathology, while the remaining cases are validated through either follow-up examinations, expert consensus, or in-vivo confocal microscopy [26].

The images in HAM10000 have the same size of 3×600×450. We center-crop the images to 3×450×450 and then resize then into five resolutions: 3×35×35, 3×75×75, 3×150×150, 3×300×300, 3×450×450, with cubic spline interpolation. The performance of MTDL on these groups are shown in Table 1. As seen in the table, MTDL achieves improved performance as the image resolution increases. This indicates that MTDL is capable of extracting more detailed features from higher-resolution inputs, which makes it well-suited for clinical applications where high-resolution images are prevalent. Notably, the best performance is achieved on the largest image resolution (3×450×450), surpassing the results obtained on the DermaMNIST dataset. Specifically, the AUC improves from 0.962 to 0.973 and the ACC improves from 0.836 to 0.863. Even at the lowest resolution (3 × 35 × 35), MTDL achieves an AUC (ACC) of 0.943 (0.797), outperforming the best existing models’ performance of 0.937 (0.780). This highlights the robust lower-bound performance of MTDL across varying data resolutions, a critical attribute for addressing real-world clinical challenges.

#### 2.2.6 Ablation Study

In our proposed MTDL model, the original image is decomposed into distinct orthogonal components, which are then concatenated to form a new composite image, serving as input to the CNN architecture. To evaluate the importance of the Hodge decomposition method, we perform an ablation study by replacing the decomposed images with the original images and denote the resulting model as ImgCNN. We compare the performance of MTDL and ImgCNN on five 2D datasets spanning different data scales, including RetinaMNIST (1,600 samples), PneumoniaMNIST (5856 samples), DermaMNIST (10,015 samples), OrganAMNIST (58,830 samples), and PathMNIST (107,180 samples). Additionally, the comparison extends to two 3D datasets: VesselMNIST3D (binary classification) and FractureMNIST3D (three-class classification). The results are summarized in Table. 2. As shown in the table, MTDL consistently outperforms ImgCNN across both 2D and 3D tasks. Notably:

- For RetinaMNIST, AUC improves from 0.838 to 0.874, and ACC improves from 0.608 to 0.655.
- For DermaMNIST, AUC improves from 0.957 to 0.962, and ACC improves from 0.808 to 0.836.
- For VesselMNIST3D, AUC improves from 0.924 to 0.937, and ACC improves from 0.903 to 0.938.

These findings demonstrate the significant potential of the Hodge decomposition approach for enhancing medical image representation, enabling improved performance across diverse datasets and classification tasks.

## 3 Discussion

TDL has achieved great success in applications involving point cloud and graph data. However, a dedicated TDL model for differentiable manifold data has not yet been developed, despite images being natural examples of such data. To bridge this gap, we introduce MTDL as a novel framework for extending TDL to differentiable manifold data. The systematic evaluation results demonstrate the efficiency, robustness, and generalizability of MTDL in medical image analysis. Additionally, our ablation studies highlight the significant potential of the Hodge decomposition approach in enhancing medical image representations.

In comparison to existing models on MedMNIST v2, MTDL is lightweight yet highly effective. For 2D datasets, the top three models in terms of average performance are MTDL, MedViT [19], and FPViT [18]. Similarly, for 3D datasets, the leading models are MTDL, C-Mixer [20], and BSDA [22]. MedViT, which combines ViT with CNN, contains over 10M parameters. FPViT uses ResNet18 for feature extraction followed by shallow ViT layers for classification, its parameters also exceed 10 M since ResNet18 alone has more than 10 M parameters. C-Mixer, a model that integrates incentive learning, a C-Mixer network, and a self-supervised pretraining framework, does not report its parameter count or provide public code. Our rough estimate suggests it exceeds 1M parameters. BSDA is a Bayesian random semantic data augmentation techniques, which can be integrated with our model. In contrast, MTDL has only 0.56M parameters for 2D tasks and 0.75M parameters for 3D tasks, which is significantly fewer than other competing models. Despite its lightweight architecture, MTDL demonstrates exceptional performance.

For topological component of MTDL, the representation of images as vector fields plays a critical role in model performance, analogous to the importance of data representation in deep learning models. While this study adopts a specific method for generating vector fields in this study, we also present alternative methods in the Supplementary Information, which warrant further investigation. For the Hodge decomposition, we employ the standard three-component decomposition method. However, the five-component decomposition, which captures richer boundary and topological information of the image manifold, represents another promising direction for future research.

For deep learning component of MTDL, the key element is a modified Transformer encoder architecture by adding a maxpooling operation and replacing the multihead attention and feedforward layers by convolutions operations. Here we deliberately use this simple architecture to highlight the topological aspects of MTDL. In follow-up studies, we plan to integrate attention mechanisms for long-range inference on medical data tensors, enabling more complex clinical tasks such as lung CT screening and diagnosis [27]. This will be explored in future work.

## 4 Methods

### 4.1 Topology-preserving Hodge Decomposition for Images

Hodge decomposition is a fundamental result in differential geometry and algebraic topology, specifically for the analysis of differential forms on Riemannian manifolds. Recently, a discrete topology-preserving Hodge decomposition for manifolds with boundaries on Cartesian grids has been introduced [12]. This method is particularly well-suited for image analysis, as images can be naturally treated as discrete manifolds with boundaries embedded in Cartesian grids.

#### 4.1.1 Hodge Decomposition in the Continuous Case

Let M be an m-dimensional smooth, orientable, compact manifold with boundary $\partial M,\Omega^{k}(M)$ represent the space of differential k-forms on M, and d denote the differential (exterior derivative) from k-forms to (k+1)-forms. A differential k-form ω is called closed if dω=0 and exact if there exists a (k−1)-form ζ such that dζ=ω.

Given a Riemannian metric g on M, let ⋆ be the Hodge star operator that maps k-forms to (m−k)-forms and (⋅,⋅) denote the induced Hodge $L^{2}$ inner product on $\Omega^{k}(M)$. The codifferential $\delta :\Omega^{k}(M)\to \Omega^{k-1}(M)$ is defined as

(1) $$\delta =(-1{)}^{m\left(k-1\right)+1}⋆d⋆.$$

A differential k-form ω is called coclosed if δω=0, and coexact if there exists a (k+1)-form ζ such that δζ=ω. The operators d and δ satisfy the following relationship

(2) $$\left(d\omega ,\eta \right)=\left(\omega ,\delta \eta \right)+\int_{\partial M}\omega \wedge ⋆\eta ,$$

where ω is a (k−1)-form, η is a k-form, and ∧ is the wedge product on differential forms. This implies that d and δ are adjoint if M is a closed manifold, i.e., ∂M=∅.

The Hodge Laplacian for differential forms is defined as

(3) Δ=dδ+δd.

The Laplacian operator maps k-forms to k-forms. The kernel of Δ is called the space of harmonic forms. We denote by ${ℋ}_{\Delta }^{k}(M)$ the space of harmonic k-forms and by ${ℋ}^{k}(M)$ the space of k-forms that are both closed and coclosed. We have ${ℋ}^{k}(M)\subset {ℋ}_{\Delta }^{k}(M)$.

When M is a closed manifold, i.e., a compact manifold without boundary. The standard Hodge decomposition [28] states that

(4) $$\Omega^{k}\left(M\right)=d\Omega^{k-1}\left(M\right)⊕\delta \Omega^{k+1}\left(M\right)⊕{ℋ}_{\Delta }^{k}\left(M\right),$$

where the adjointness of d and δ ensures that these three subspaces are orthogonal with respect to the Hodge $L^{2}$ inner product.

When M is a manifold with non-empty boundary, the operators d and δ are generally not adjoint, as noted in (2). To ensure their adjointness and consequently achieve an orthogonal decomposition of differential forms, appropriate boundary conditions must be imposed.

Two most commonly used boundary conditions are the normal (Dirichlet) boundary condition and the tangential (Neumann) boundary condition. These conditions define the following subspaces,

(5) $$\Omega_{n}^{k}\left(M\right)=\left\{\omega \in \Omega^{k}(M)|\;\omega {|}_{\partial M}=0\right\},\;\Omega_{t}^{k}\left(M\right)=\left\{\omega \in \Omega^{k}(M)|\;⋆\omega {|}_{\partial M}=0\right\}.$$

The forms in $\Omega_{n}^{k}(M)$ and $\Omega_{t}^{k}(M)$ are called normal and tangential respectively.

The Hodge-Morrey decomposition [29] states that

(6) $$\Omega^{k}\left(M\right)=d\Omega_{n}^{k-1}\left(M\right)⊕\delta \Omega_{t}^{k+1}\left(M\right)⊕{ℋ}^{k}\left(M\right).$$

The exterior derivative d preserves the normal boundary condition and the codifferential δ preserves the tangential boundary condition. As a result, any k-form can be decomposed as the sum of an exact normal form, a coexact tangential form, and a harmonic form that is both closed and coclosed.

(7) $$\omega =d\alpha_{n}+\delta \gamma_{t}+\eta ,$$

where $\omega \in \Omega^{k}(M)$, $\alpha_{n}\in \Omega_{n}^{k-1}(M)$, $\gamma_{t}\in \Omega_{t}^{k+1}(M)$, $\eta \in {ℋ}^{k}(M)$. When we focus on the compact manifold in Euclidean spaces, the third term ${ℋ}^{k}(M)$ in (6) can be further decomposed into three orthogonal components [30], resulting in a five-component decomposition. A more detailed description of the Hodge decomposition can be found in the Supplementary Information.

#### 4.1.2 Discrete Topology-preserving Hodge Decomposition for Medical Images

A medical image can be naturally seen as a level set function on a Cartesian grid, with its pixel values defining the scalar field. This makes discrete Hodge decomposition on Cartesian grids particularly suitable for the analysis of medical images.

Here we focus on 2D and 3D Cartesian grids, as medical images are typically in these dimensions. The discrete manifold M on Cartesian grids can be given as a sublevel set of a level set function on the grid. We employ the strategy in [31] to determine the boundary of M for two boundary conditions. For normal boundary condition, cells with at least one vertex inside M are included, while for tangential boundary condition, cells with at least one vertex of their dual cells inside M are included. The resulting sets of cells are referred to as the normal support for the normal boundary condition and the tangential support for the tangential boundary condition. These supports can be seen as discrete versions of the manifolds with boundary. The boundary of M is typically detected using a projection matrix. The projection matrices $P_{k,n}$ and $P_{k,t}$ for normal and tangential boundary conditions are derived from the identity matrix by removing rows corresponding to cells outside the respective supports.

On a Cartesian grid, vertices, edges, faces, and cubes are referred to as 0-cells, 1-cells, 2-cells and 3-cells. A differential k-form can be discretized as a k-cochain, which is a real-valued function on the k-cells. For instance, an image can be seen as a discrete 0-form since it is a 0-cochain on the Cartesian grid. The differential operators, including exterior derivative, Hodge star, codifferential, and Laplacian, can be discretized as matrices. Formally, let $I_{m}$ be a Cartesian grid with cells oriented according to the coordinate axes, and $D_{k}$ denote the discrete exterior derivative on ${\text{I}}_{m}$, then the discrete exterior derivative on M for normal and tangential boundary conditions, denoted by $D_{k,n}$ and $D_{k,t}$ are

(8) $$D_{k,n}=P_{k+1,n}D_{k}P_{k,n}^{T},\;D_{k,t}=P_{k+1,t}D_{k}P_{k,t}^{T}.$$

Let $S_{k}$ denote the discrete Hodge star on $I_{m}$, the discrete Hodge star on M for normal and tangential boundary conditions are $S_{k,n}$ and $S_{k,t}$ respectively as follows

(9) $$S_{k,n}=P_{k,n}S_{k}P_{k,n}^{T},\;S_{k,t}=P_{k,t}S_{k}P_{k,t}^{T}.$$

With the discrete Hodge star and discrete exterior derivative, the discrete codifferential can be expressed as $S_{k-1,n}^{-1}D_{k-1,n}^{T}S_{k,n}$ and $S_{k-1,t}^{-1}D_{k-1,t}^{T}S_{k,t}$ for normal and tangential boundary conditions respectively. The discrete Hodge Laplacian for normal and tangential boundary conditions $L_{k,n}$ and $L_{k,t}$ respectively are as follows

(10) $$L_{k,n}=D_{k,n}^{T}S_{k+1,n}D_{k,n}+S_{k,n}D_{k-1,n}S_{k-1,n}^{-1}D_{k-1,n}^{T}S_{k,n},\;L_{k,t}=D_{k,t}^{T}S_{k+1,t}D_{k,t}+S_{k,t}D_{k-1,t}S_{k-1,t}^{-1}D_{k-1,t}^{T}S_{k,t}.$$

As in the continuous case, the Kernels of these discrete Laplacians are fully determined by the topology of M. Specifically, the dimension of $\text{ker}L_{k,n}$ equals the Betti number $\beta_{m-k}$, while the dimension of $\text{ker}L_{k,t}$ equals $\beta_{k}$. The Betti number $\beta_{k}$ quantifies the number of k-dimensional topological features in $M:\beta_{0}$ represents the number of connected components, $\beta_{1}$ the number of loops, and $\beta_{2}$ the number of voids.

**Figure 4:**
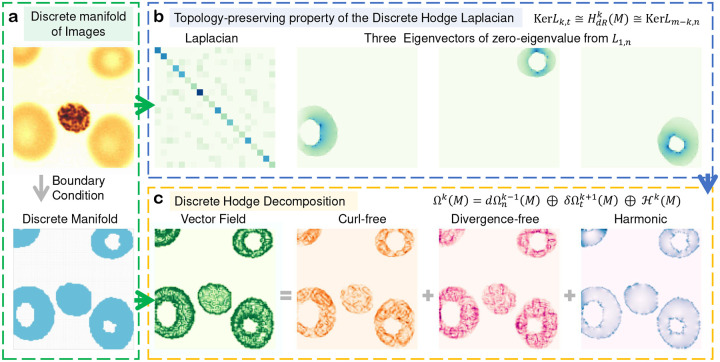
Illustration of the topology-preserving property of the discrete Hodge Laplacian and the Hodge decomposition for a medical image. In (**a**), the foreground of the image is represented as a manifold with boundary. The Laplacian $L_{1,n}$ is computed and its eigenvectors corresponding to the zero eigenvalues are displayed, accurately capturing the three loops in the manifold (**b**). In (**c**), a vector field (1-form) is constructed from the image and decomposed into three orthogonal components: the curl-free, divergence-free, and harmonic parts. The harmonic component encapsulates the global topological information, while the other two components convey distinct aspects of local information.

Fig. 4 illustrates an example demonstrating the topology-preserving property of the discrete Laplacian. As shown in the figure, a blood cell image is represented as a discrete manifold under boundary conditions (Fig. 4a). This manifold exhibits three distinct loop structures, resulting in a Betti number $\beta_{1}$ of 3. We compute the Laplacian $L_{1,n}$ under the normal boundary condition, and the eigenvectors corresponding to the three zero eigenvalues are displayed. These eigenvectors align precisely with the three loops present in the manifold (Fig. 4b).

With the discrete versions of differential forms and operators established, the discrete Hodge decomposition is expressed as:

(11) $$V^{k}=D_{k-1,n}W_{n}+S_{k,t}^{-1}D_{k,t}^{T}S_{k+1,t}W_{t}+E,$$

where $V^{k},W_{n},W_{t}$, and E are the discrete version of $\omega ,\alpha_{n},\beta_{t}$, and η in (7) respectively.

Fig. 4 illustrates an example of the Hodge decomposition applied to a blood cell image. As shown in Fig. 4c, a vector field (1-form) on the manifold is first derived from the image using the flow-based method described in Supplementary Information. This vector field is subsequently decomposed into three orthogonal components: the curl-free, divergence-free, and harmonic parts. The harmonic component represents the global topological structure of the underlying manifold, whereas the normal and tangential components characterize distinct aspects of the local information. Specifically, the textures of the normal and tangential components exhibit an approximately perpendicular relationship, and the harmonic component appears smoother compared to the other two components.

### 4.2 CNN Architecture

The CNN we used is based on the Transformer encoder architecture by adding a maxpooling operation and replacing the multihead attention and feedforward layers by convolution operations (Fig. 1e).

As illustrated, the decomposed image x is first processed through an initialization block, which consists of a convolutional layer, a batch normalization operation, and a nonlinear ReLU activation function. The initialized image is then passed through a sequence of Transformer-encoder-induced convolution layers to extract hierarchical features. Finally, the extracted features are spatially averaged and fed into a multilayer perceptron (MLP) for classification.

A Transformer-encoder-induced convolution layer is composed of two convolutional blocks followed by a pooling operation. Formally, for an input image x, the TransConv layer is defined as:

(12) $$x^{\prime }=\text{Norm}\left(x+\text{MultiHeadConv}\left(x\right)\right),\;x^{\prime \prime }=\text{Norm}\left(x^{\prime }+\text{FeedForwardConv}\left(x^{\prime }\right)\right),\;x^{\prime \prime \prime }=\text{MaxPool}\left(x^{\prime \prime }\right),$$

where Norm is the batch normalization operation, MaxPool represents the max pooling operation, and $x^{\prime \prime \prime }$ is the output for x after a TransConv layer. If the input x is a 2D image of dimensions (W,H,C), where W,H and C correspond to the width, height, and number of channels, respectively, then the output $x^{\prime \prime \prime }$ will have dimensions $\left(\frac{W}{2},\frac{H}{2},C\right)$ due to the pooling operation.

The MultiHeadConv block is designed to mimic the multi-head attention mechanism in the Transformer encoder. It consists of a group convolution, a ReLU activation, and a 1×1 convolution operation. Let ${\text{Conv}}_{C_{\text{in}},C_{\text{out}},k,g}$ denote a convolution operation with a kernel size k, group number g, input channel $C_{\text{in}}$, and output channel $C_{\text{out}}$. For an input image x with C channels, the MultiHeadConv block is expressed as

(13) $$x^{\prime }={\text{Conv}}_{C,C\times h,3,h}(x)\;x^{\prime \prime }=\text{ReLU}\left(x^{\prime }\right)\;x^{\prime \prime \prime }={\text{Conv}}_{C\times h,C,1,1}\left(x^{\prime \prime }\right)$$

where $x^{\prime \prime \prime }$ is the output of x after the MultiHeadConv block, h is a hyperparameter corresponding to the number of heads in the multi-head attention mechanism. The first convolution emulates the multi-head attention operation, while the second 1×1 convolution serves as a linear layer for feature fusion. Importantly, the MultiHeadConv block preserves the input image dimensions.

The FeedForwardConv block imitates the feedforward neural network layers typically found in the Transformer’s encoder. It consists of two group convolutions separated by a ReLU activation function. Formally, for an input image x with C channels, the FeedForwardConv block is defined as:

(14) $$x^{\prime }={\text{Conv}}_{C,2\times C,1,g}(x),\;x^{\prime \prime }=\text{ReLU}\left(x^{\prime }\right),\;x^{\prime \prime \prime }={\text{Conv}}_{2\times C,C,1,g}\left(x^{\prime \prime }\right),$$

where the two 1 × 1 convolutions mimic the linear layers in a standard feedforward neural network. Similar to the MultiHeadConv block, the FeedForwardConv block maintains the input image dimensions.

### 4.3 Model Implementation Detail

#### 4.3.1 Decomposed Image Generation

In our implementation, each image is considered as a scalar field on the vertices of a standard Cartesian grid. The discrete manifold is generated by a segmentation, which involves extracting the foreground pixels by applying a threshold to remove background pixels from the images. We use the grid vertices, edges, faces, and cubes to construct the differential operators and projection operators in Sec. 4.1.2.

Instead of taking the differential operator directly on the scalar field to construct the 1-form ω. We instead follow a 2-step procedure to provide noise resilience. First, we use the discrete gradient operation to get a vector field stored on the vertices. Formally, For a 3D image ℐ, where ℐ(i,j,k) represents the pixel value at position (i,j,k), a vector $\left(x_{i,j,k},y_{i,j,k},z_{i,j,k}\right)$ for the pixel at (i,j,k) is constructed by the following centered finite differences

(15) $$x_{i,j,k}=\frac{ℐ(i+1,j,k)-ℐ(i-1,j,k)}{2},\;y_{i,j,k}=\frac{ℐ(i,j+1,k)-ℐ(i,j-1,k)}{2},\;z_{i,j,k}=\frac{ℐ(i,j,k+1)-ℐ(i,j,k-1)}{2}.$$

Second, this vector field is averaged into a 1-form ω on the edges. Let $e_{i,j,k}^{x}$ denote the edge connecting the vertices at (i,j,k) and (i+1,j,k), $e_{i,j,k}^{y}$ denote the edge connecting the vertices at (i,j,k) and (i,j+1,k), and $e_{i,j,k}^{z}$ denote the edge connecting the vertices at (i,j,k) and (i,j,k+1). The 1-form ω is defined as


(16) $$\omega \left(e_{i,j,k}^{x}\right)=\frac{x_{i,j,k}+x_{i+1,j,k}}{2},\;\omega \left(e_{i,j,k}^{y}\right)=\frac{y_{i,j,k}+y_{i,j+1,k}}{2},\;\omega \left(e_{i,j,k}^{z}\right)=\frac{z_{i,j,k}+z_{i,j,k+1}}{2}.$$

Finally, following the decomposition described in (7), the 1-form ω is decomposed into three orthogonal components

(17) $$\omega =\omega_{1}+\omega_{2}+\omega_{3}.$$

Here the decomposition is performed by the BIG Laplacian. For each component 1-form η resulting from this decomposition, it is represented as a vector field stored on grid cubes. For the cube with the lowest indexed corner at position (i,j,k), the corresponding vector $\left(\eta_{i,j,k}^{x},\eta_{i,j,k}^{y},\eta_{i,j,k}^{z}\right)$ is given by averaging its projection on an axis direction along 4 edges in that direction:

(18) $$\eta_{i,j,k}^{x}=\left[\eta \left(e_{i,j,k}^{x}\right)+\eta \left(e_{i,j+1,k}^{x}\right)+\eta \left(e_{i,j,k+1}^{x}\right)+\left.\eta \left(e_{i,j+1,k+1}^{x}\right)\right]/4\right.\;\eta_{i,j,k}^{y}=\left[\eta \left(e_{i,j,k}^{y}\right)+\eta \left(e_{i+1,j,k}^{y}\right)+\eta \left(e_{i,j,k+1}^{y}\right)+\right.\left.\eta \left(e_{i+1,j,k+1}^{y}\right)\right]/4\;\eta_{i,j,k}^{z}=\left[\eta \left(e_{i,j,k}^{z}\right)+\eta \left(e_{i+1,j,k}^{z}\right)+\eta \left(e_{i,j+1,k}^{z}\right)+\right.\left.\eta \left(e_{i+1,j+1,k}^{z}\right)\right]/4$$

The resulting vector field η can be interpreted as a three-channel image, with each channel corresponding to one of the x,y and z-axes. Finally, we concatenate the three-channel images derived from the three components in (17) to construct a nine-channel image, which serves as the final decomposed representation. For 2D images, a similar procedure is applied, using only the x and y-components in equations (15), (16), (17), and (18) to obtain the decomposed representations.

### 4.3.2 Model details

The proposed MTDL model is implemented using PyTorch [32] and evaluated on an NVIDIA Tesla V100S GPU. For 2D datasets, the batch size and learning rate are set to 64 and 10^−3^, respectively, across all tasks. The training process spans 30 epochs for tasks with a sample size smaller than 100,000 and 10 epochs for tasks with a sample size exceeding this threshold. The number of layers in the model is adapted based on the data distribution. For the majority of tasks, a 5-layer structure is employed, with detailed configurations provided in the Supplementary Information. The hidden channel dimension C and head number h are set to 72 and 4, with the group number g configured as 1 for grayscale images and 3 for colored images.

For 3D datasets, the batch size and learning rate are set to 16 and 10^−3^, respectively, for all tasks. The training process involves 10 epochs for the FractureMNIST dataset and 20 epochs for the remaining datasets. Similar to the 2D case, the number of layers is determined by the data distribution, with further details available in the Supplementary Information. The hidden channel dimension C and head number h are set to 64 and 4, with a group number g of 1 since all the 3D images are grayscale.

The model is optimized using the AdamW optimizer [33] with a weight decay of 10^−5^, and a one-cycle learning rate scheduler employed [34]. For the ChestMNIST2D task, a multi-label classification problem, the Binary Cross-Entropy with Logits is used as the loss function, while Cross-Entropy Loss is applied for all other tasks.

## Figures and Tables

**Table 1:** Performance of MTDL over different image reslutions

Resolution	3 × 35 × 35	3 × 75 × 75	3 × 150 × 150	3 × 300 × 300	3 × 450 × 450
AUC	0.943	0.947	0.958	0.970	0.973
ACC	0.797	0.803	0.822	0.856	0.863

**Table 2:** Performance Comparison between the original images and decomposition images, the best result is in bold.

Methods	ImgCNN		MTDL	
	AUC	ACC	AUC	ACC
RetinaMNIST	0.838	0.608	**0.874**	**0.655**
PneumoniaMNIST	0.978	0.885	**0.986**	**0.910**
DermaMNIST	0.957	0.808	**0.962**	**0.836**
OrganAMNIST	**0.998**	0.955	**0.998**	**0.956**
PathMNIST	0.987	0.902	**0.996**	**0.920**
VesselMNIST3D	0.924	0.903	**0.937**	**0.938**
FractureMNIST3D	0.749	0.566	**0.753**	**0.583**

## Data Availability

The data used in this study can be found on the MedMNIST official website medmnist.com.
